# Counter-attack of biocontrol agents: Environmentally benign Approaches against Root-knot nematodes (*Meloidogyne* spp.) on Agricultural crops

**DOI:** 10.1016/j.heliyon.2023.e21653

**Published:** 2023-10-28

**Authors:** Amir Khan, Mohammad Haris, Touseef Hussain, Abrar Ahmad Khan, Salah-Eddine Laasli, Rachid Lahlali, Fouad Mokrini

**Affiliations:** a Plant Pathology and Nematology Section, Department of Botany, Aligarh Muslim University, Aligarh, 202002, UP, India; b Section of Environmental Botany, Department of Botany, Aligarh Muslim University, Aligarh, 202002, UP, India; c Division of Plant Pathology, ICAR-Indian Agricultural Research Institute, New Delhi, 110012, India; d Phytopathology Unit, Department of Plant Protection, Ecole Nationale d’Agriculture de Meknès, Km10, Rte Haj Kaddour, BP S/40, Meknès, 50001, Morocco; e Plant Pathology Laboratory, AgroBioSciences, College of Sustainable Agriculture and Environmental Sciences, Mohammed VI Polytechnic University Lot 660, Hay Moulay Rachid Ben Guerir, 43150, Morocco; f Biotechnology Unit, Regional Center of Agricultural Research, INRA-Morocco, Rabat, Morocco

**Keywords:** Root-knot nematodes, Parasitic proteins, Bio-control, Nematophagous fungi, Trapping devices, Bacterial antibiosis, Pathogenesis-related genes, Induced systemic resistance

## Abstract

Root-knot nematodes (*Meloidogyne* spp.) are obligate sedentary endoparasites, considered severe crop-damaging taxa among all plant-parasitic nematodes globally. Their attacks through parasitic proteins alter the physiology and machinery of the host cells to favour parasitism and reduction in crop yield. Currently, the use of excessive pesticides as a fast remedy to manage this pest is hazardous for both the environment and humans. Keeping this view in mind, there is an urgent need for developing efficient eco-friendly strategies. Bio-control as an eco-friendly is considered the best approach to manage nematodes without disturbing non-target microbes. In bio-control, living agents such as fungi and bacteria are the natural enemies of nematodes and the best substitute for pesticides. Fungi, including nematode-trapping fungi, can sense host signals and produce special trapping devices *viz*., constricting rings and adhesive knobs/loops, to capture nematodes and kill them. Whereas, endo-parasitic fungi kill nematodes by enzymatic secretions and spore adhesion through their hyphae. Bacteria can also control nematodes by producing antibiotic compounds, competing for nutrients and rhizosphere, production of hydrolytic enzymes *viz*., chitinases, proteases, lipases, and induction of systemic resistance (ISR) in host plants. Scientists throughout the world are trying to evolve environmentally benign methods that sustain agricultural production and keep nematodes below a threshold level. Whatever methods evolve, in the future the focus should be on important aspects like green approaches for managing nematodes without disturbing human health and the environment.

## Highlights

• Plant-parasitic root-knot nematodes are the major soil-borne pests for agricultural production.
• Biocontrol mechanisms as eco-friendly against such pests become the emerging approaches.
• In bio-control mechanisms living agents and their products stimulate the resistance genes in plants without disturbing beneficial soil microbes.
• Biocontrol agents and their metabolic products also stimulate systemic resistance in plants.

## 1 Introduction

The rhizosphere is the residence of several microbes that interact with each other and the plant roots. Some of them are advantageous for plants and soil microbes while others are deleterious. Such deleterious microbes are considered pests that incite biotic stress and lower the productivity of affected crops [1]. Plant-parasitic nematodes (PPNs) are one of the most serious plant-damaging soil microbes for agricultural production worldwide [2,3]. Currently, around 4100 species of PPNs have been discovered that affect various crops like fruits including grapevine, bananas, sugarcane, and vegetables *viz*., brinjal, chili, tomatoes, cauliflower, cabbage, carrot, okra, maize, cotton, potatoes and soybeans [4,5]. The impact of PPNs is more observed in vegetable-producing countries *viz*., Bangladesh, India, Pakistan, and some areas of African countries where the major issues are poverty, hunger, and inadequate food supply. According to nematologists, the top 10 PPNs can significantly damaging global agriculture production; these are root-knot nematodes (*Meloidogyne* spp.), root lesion nematode (*Pratylenchus* spp.), cyst nematodes (*Heterodera and Globodera*), pine wilt nematode (*Bursaphelenchus xylophilus*), burrowing nematode (*Radopholus similis*), rice white tip nematode (*Aphelenchoides besseyi*), reniform nematode (*Rotylenchulus reniformis*), stem and bulb nematode (*Ditylenchus dipsaci*), dagger nematode (*Xiphinema index-*the virus vector nematode) and false root-knot nematode (*Nacobbus aberrans*) [6]. However, root-knot nematodes (*Meloidogyne* spp.) are the most important and dangerous soil-borne pathogen among all PPNs (Khan et al., 2023).

## 2 Biotic stress

Biotic stress is the response of phytopathogenic infection, especially root-knot nematodes (RKNs), fungi, and bacteria that bring down productivity and crop yield [7]. Such harmful pests *viz*., RKNs, acquire their nutrition from the host plant and cause the weakening and killing of host cells [8]. Plants' reply to such biotic stress is complicated, involving interaction between two living organisms. Simultaneously, plants evolve protective and defensive mechanisms against such pathogenic interactions. Thus, pathogen-plant interaction is a multifaceted method where actual interaction takes place between plant and pathogen-derived stimuli *viz*., proteins, genes, sugars and lipopolysaccharides. Such stimuli obtain from pathogens are responsible for the severity of infection and pathogenicity which is determined by the pathogen's ability to colonize the plant cell [9].

## 3 Root-knot nematodes (*Meloidogyne* spp.) as a major biotic stress-causing agent

RKNs (*Meloidogyne* spp.) are major biotic stress-causing agents for several crops globally. It belongs to the order of Tylenchida, ubiquitous in nature and sedentary endoparasites that depend on the host for completing their life cycle [10]. More than 100 species of RKNs have been discovered that affect almost 3000 plants, including vegetables and fruits, globally [11]. Among them, four species of RKNs *viz*., *M. javanica, M. incognita, M. hapla* and *M. arenaria* are major and harmful, causing more damage up to 90 % to the plants and making them more susceptible to other pathogens [12,13]. Fourteen root-knot species have been recognized from distinct places in India [14]. Out of which, *M. incognita* is one of the more damaging species among all, causing major loss and reduction in vegetables yield and quality *viz*., tomato, okra, eggplant, cauliflower, cabbage and spinach [15]. However, it has been also observed that certain fields in India were predominantly occupied by root-knot disease due to which vegetable crops were affected severely (Khan et al., 2022). Other species *viz*., *M. graminicola, M. javanica,* and *M. arenaria* are also pathogenic and common [14]. However, *M. hapla* is restricted to temperate/cooler areas [16] (Table 1).

**Table 1:** Worldwide losses on agricultural crops due to root-knot nematodes.

Root-knot nematode species	Host crop	Losses (%)	Country	Authors
*Meloidogyne incognita*	Tomato	40 %	India	[17]
*Meloidogyne* spp	Tomato	24–38 %	Pakistan	[18]
*Meloidogyne* spp	Tomato	30 %	Nepal	[19]
*Meloidogyne* spp	Tomato	80 % approx	Turkey	[20]
*Melodogyne* spp	Chilli	8–23 %	India	[21]
*M. incognita*	Ivy gourd	35.09 %	India	[22]
*Melodogyne* spp	Radish	8–20 %	India	[21]
*M. graminicola*	Rice	42 %	Southeast Asia	[23]
*M. graminicola*	Rice	37–45 %	India	[15,24]
*M. incognita*	Brinjal	16–41.8 %	Pakistan (Punjab)	[25]
*M. incognita*	Brinjal	16.67 %	India	[26]
*M. incognita*	Carrot	10 %	India	[26]
*M. incognita*	Chickpea	19–40 %	India	[27]
*M. javanica*	Chickpea	24–61 %	India	[27]
*M. arenaria, M. javanica*	Groundnut	21.60	India	[26]
*M. incognita*	*Pomegranate*	17.30	India	[26]
*M. incognita*	Jute	21.35	India	[26]
*Meloidogyne* spp.	Cucumber	52	Pakistan	[28]

## 4 Biology of root-knot nematodes: the journey from soil to host

**Fig. 1:**
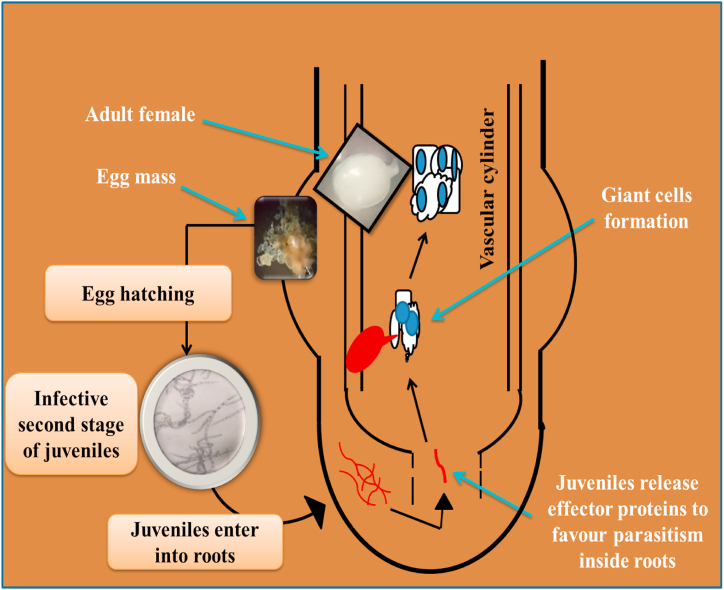
Biology of root-knot nematodes (*Meloidogyne* spp.).

**Fig. 2:**
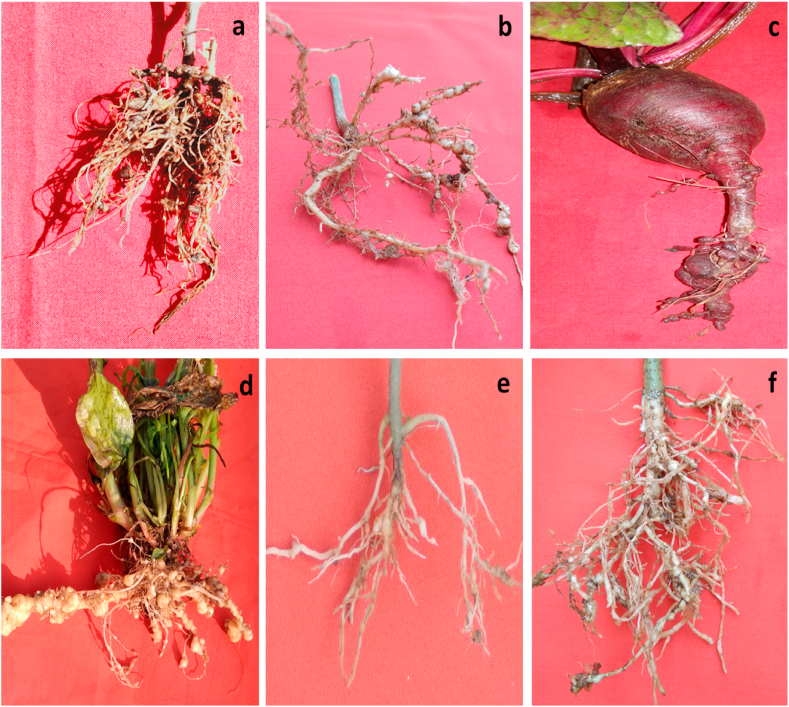
Galled root samples of various agricultural crops infected with root-knot nematodes, **(a)** root of chickpea, **(b)** root of black gram, **(c)** beet root, **(d)** spinach root, **(e)** tomato root, and **(f)** okra root.

RKNs exhibited sexual dimorphisms, i.e., the females are pear-shaped and the males are free-living while second-stage juveniles (J2s) are infective. The life cycle of the RKNs is completed in 25–28 days at 27 °C temperature and divided into different stages (*viz*., eggs, juveniles, and adults) but this period is changed due to soil moisture (lesser extent), soil temperature (widely) and availability of a suitable host [11] (Fig. 1). The successful completion of the life cycle, involving sequential molts from egg to adult, includes morphologically and functionally distinct stages. In optimal conditions, a single female can produce 200–500 eggs in a mucilaginous matrix [11]. Embryogenesis converts eggs into J1s, which remain inside the eggshell. Following the first molt, J1 transforms into J2s, which comes out in the soil from the eggshell to search for a host plant [29]. By using chemosensory amphids, J2s migrate toward the susceptible host roots by sensing chemical gradients secreted by the roots [30]. During the compatible interaction with the susceptible plants, J2s penetrate the roots and migrate straight down, between the cortical cells, to the apical meristematic site. Then they start moving upwards in the vascular bundle, inducing specialized hypertrophied feeding cells known as giant cells (GCs). These GCs undergo multiple mitotic cycles without cytokinesis, which causes them to enlarge and become multinucleated [31]. GCs are the places, where nematodes complete their life cycle and occupy sedentary habitats [32]. They possess a hollow, protruding stylet at their anterior end, which they utilize to inject secretions into and extract nutrients from GCs. The hyperplasia of the root cells leads to irregular growth and causes the formation of galls or root-knot, which is a unique feature of root-knot disease caused by RKNs [33] (Fig. 2).

## 5 Effector proteins of root-knot nematodes: favour parasitism by altering the physiology and metabolism of the host cells

Effector proteins of RKNs are pathogenic in nature that parasitize the host plant by changing the metabolism and physiology of the host cell. Parasitism and manipulation of host cellular mechanisms are favoured by the interactions of effector proteins with certain host proteins. Such interactions *viz*., parasitic proteins-host proteins also suppress and weaken the defence and defence-related genes of the host [34]. During parasitism, J2s release oesophageal parasitic proteins through their stylet (piercing organ) into the host tissues and induce GCs formation [35]. However, J2s release such parasitic proteins either on the cell surface or directly within the cytoplasm of the host cell. Both of the cases mimic the few proteins that result in the changes in the expression of host cell genes [36]. Effectors proteins, cell wall digesting enzymes (CWDEs), and the proteins that mimic the proteins of the host are important secretions of RKNs for the successful feeding and their establishment on the host [37,38] (Table 2).

**Table 2:** List of some important and novel effector proteins secreted by root-knot nematodes to favour parasitism within the host plant.

Root-knot nematode species	Effector proteins of RKNs	Function	Test crop	Authors
*M. arenaria*	Hsp40 (DnaJ)	Host cell remodeling	Tomato	[39]
*M. graminicola*	MgPDI	Attack on host plant ROS system	Rice	[40]
*M. incognita*	MiISE6	Targets the host nucleus	*Arabidopsis thaliana*	[41]
*M. incognita*	Mi IDL1	Formation of galls	*Arabidopsis* plant	[37]
*M. incognita*	MiMIF	Suppression of annexin mediated immune response	*Arabidopsis* plant	[42]
*M. incognita*	Mi-asp2	Predigestion of peptidic nutrients	*Arabidopsis thaliana*	[43]
*M. incognita*	16D10 peptide	Excessive root growth stimulation for nematode feeding	*Arabidopsis* plant	[44]
*M. javanica*	Chorismate mutase-1 (MjCM-1)	Causes Auxin deficiency and alters plant cell development	Soybean	[45]
*Meloidogyne* spp.	Pectate lyase-like genes (PLL18 and PLL19)	Degradation of cell wall	*Arabidopsis*	[46]
*M. javanica*	Mj TTL 5	Suppress host immune response	*Arabidopsis*	[47]
*M. incognita*	Mi-CM-3	Suppressing the plant immunity by manipulating the salicylic acid pathway	*Nicotiana benthamiana*	[48]
*M. graminicola*	MgMO237	Suppress host defence responses	Rice	[49]
*M. incognita*	MiMsp40	Inhibit host immune response	*Arabidopsis* plant	[50]
*M. incognita*	Mi-CRT (calreticulin)	Suppress host defences	*Arabidopsis* plant	[51]
*M. graminicola*	Mg16820	Suppresses both pathogens triggered immunity effector triggered immunity	*Nicotiana benthamiana*	[52]
*M. incognita*	MiPFN3	Disrupts plant actin filaments and promotes parasitism	*Arabidopsis* plant	[53]
*M. incognita*	MiIDL1	Favour parasitism in host plant	*Arabidopsis* plant	[37]
*M. javanica*	MjTTL5	Suppress plant immune response	*Arabidopsis plant*	[47]

### 5.1 Modes of action of effector proteins

The cell wall of the host plant acts as the first barrier for the entry of any pathogen, including RKNs. For the successful establishment and crossing of such a barrier, RKNs release several CWDEs for the damaging cellular constituents of the host cell. In addition to CWDEs, RKNs release a few other enzymes to favour parasitism within the host *viz*., polygalacturonase, pectate lyases, 1, 4 β endoglucanase, endo 1,4 β xylanase, expansins, and a cellulose binding protein [35,[54], [55], [56], [57]]. Some of the effector proteins are released by J2s through their three oesophageal glands including two sub ventral glands [58]. The recipient of the secretory substances of RKNs is located in the apoplasm of the host plant [43].

#### 5.1.1 Inhibition of the defence-related plant hormones of the host

Plant hormones such as auxin (indole acetic acid, IAA), ethylene, jasmonic acid (JA), cytokinin and salicylic acid (SA) play an important role in the growth and development of plants. Several functions like cell differentiation, elongation, cell expansion and response to various biotic and abiotic stresses are mediated by ethylene and auxin [59]. Such hormones play an impressive role in phytopathogenic interactions. An effector, Mi-CM is released by *M. incognita* that changed the auxin pool of the host plant [60]. Recently, a few researchers reported that *M. incognita* possess an effector protein MiISE6 engaged in the plant-nematode interaction. This effector protein plays a major role during the initial stages of parasitism by *M. incognita* and obstructs several signalling pathways *viz*., JA pathways of the host plant [41]. Another parasitic protein Mi-CM-3 was also found in *M. incognita* which inhibits the SA pathway and plant immune system of the host plant and favours parasitism within the host [48].

#### 5.1.2 Suppression of effector-triggered immunity and/or pathogen-triggered immunity of the host

Few effector proteins *viz*., MiMsp40 from *M. incognita* suppress the immune system *viz*., effector-triggered immunity (ETI) and pathogen-triggered immunity (PTI) of the host plant and enhance nematode parasitism [50]. Mi-CRT (calreticulin) is another effector released by nematodes into the apoplasm of the host tissue that causes the inhibition of the PTI triggered by the PAMP elf8 in *Arabidopsis thaliana* [51].

#### 5.1.3 Mimicry and manipulation of the defence-related proteins of the host

*Meloidogyne graminicola* is a very harmful nematode for rice growth and brings down rice production. An important effector protein, Mg16820 obtained from *M. graminicola*, reacts as a suppressor of the immune system of the host cell and shows an effective role in nematode parasitism. Furthermore, Mg16820 inhibits flg22-induced reactive oxygen species (ROS) in the rice plant that participate in the stress response and also suppress the R2/Avr2-and Mi-1.2-induced hypersensitive response [52]. One more effector named, MiPFN3 (*M. incognita* Profilin 3) was obtained from J2s of *M. incognita*. MiPFN3 is inoculated within the host plant through the stylet of *M. incognita* where it favours and stimulates nematode parasitism [53]. MiIDL1 (*M. incognita* IDA – like 1 effector) a potent effector protein of *M. incognita* that mimic with functional IDA (Inflorescence deficient in abscission) or IDA like genes of *Arabidopsis* for favouring parasitism in *Arabidopsis* plant in the form of root galls [37]. Lin et al. [47], found an effector MjTTL5 from *M. javanica* that speeds up parasitism by attacking the ferredoxin/thioredoxin system of the host.

#### 5.1.4 Changes in the host cell metabolism

GCs formation in host roots by RKNs influence the metabolic mechanisms of the host plant because such cells act as feeder cells and source of nutrients and metabolites for developing nematodes. Few cells in the vicinity of GCs also become large due to the accumulation of huge amounts of nutrients. Such neighbouring cells provide a direct approach for food to RKNs. Eloh et al. [61] observed the changes in metabolic pathways when plants are infected with RKNs. The latter's infection also changes the methods of protein formation in host roots [62].

By examining these all changes in the host's cell physiology and metabolism, including translation it has been necessary to develop effective eco-friendly management strategies against RKNs.

## 6 Bio-control methods against root-knot nematodes parasitism

The intensity for selecting microbes as bio-control agents (BCAs) has grown day by day due to their pest-managing proprieties becoming a key factor in integrated nematode management (INM) on crops [63]. The rhizosphere harbours numerous microbes which provide resistance to plants against several pathogens, including RKNs [64]. However, the bio-control of RKNs has been researched for more than a decade. It might be recognized as a multi-trait wonder whose achievement relies upon the rhizospheric competition and interactions with other microbes, the adjustment in different environmental situations and more defence of the plant against several pathogens including RKNs [65,66]. Uses of chemical-based pesticides are the fastest methods to control plant disease however it is very noxious to the environment, human being and soil also decrease soil fertility and make soil disintegrate. So, there is a need to find out substitutes for pesticides to control plant diseases caused by PPNs. Bio-control is considered as a good alternative for chemical-based nematicides and manages plant disease in an eco-friendly manner and also increases soil fertility without disturbing fauna and flora [67,68]. Among bio agents, fungi (arbuscular mycorrhizal, nematophagous fungi, and *Trichoderma* spp.) and bacteria are considered potent bioagents against RKNs and other PPNs. Besides whole microbes sometimes derivatives *viz*., microbial metabolites and their products can also utilize as BCAs [69,70].

## 7 Fungi-mediated bio-control against root-knot nematodes

Fungi and their metabolites are found to be control the population of PPNs, which are detrimental to plant health. *Trichoderma* spp. well-known individuals recognized as important BCAs against RKNs, parasitize the infective juveniles and inhibit root penetration by nematodes also enhance the growth and yield of crops [71]. To prevent the crop from nematode infestation *Trichoderma* spp. must be applied in the soil before crop planting because it completely colonizes the roots [72]. However, the addition of organic matter to soil (e.g., chicken litter) can enhance the bio-control activity of *Trichoderma* [73]. Other groups’ *viz*., nematophagous and arbuscular fungi also possess nematicidal activity against RKNs. The bio-control activity of BCAs depends on the nematode species, host plant, its exudates, and other rotation crops [74].

### 7.1 Nematophagous fungi as bio-control agents and their modes of action

Fungi that feed on PPNs are considered as nematophagous fungi. These fungi are classified into four groups based on the mechanisms of action against nematodes: (i) predatory (nematode trapping) fungi make extensive constricting rings and hyphal networks as trapping tools for capturing nematodes, eg., *Arthrobotrys oligospora* and *Drechslerella* sp., (ii) endoparasitic fungi (obligate parasites of nematodes) parasitize nematodes either by direct ingestion or attaching to their surface followed by growth, germination, and killing of nematodes, eg., *Drechmeria coniospora*, (iii) nematode's eggs and female parasitic fungi, which as facultative parasites of sedentary stages of nematodes such as cysts and eggs, eg. *Pochonia chlamydosporia* (*Metacordyceps chlamydosporia*)*, P. rubescens* and *Paecilomyces lilacinus* and (iv) toxins producing fungi inhibit the nematodes mobility before hyphae penetrate the nematode cuticle, eg. *Pleurotus ostreatus* [75,76]. However, few nematophagous fungi exist as facultative saprotrophs, *i.e*., taking their nutrition from dead and decaying organic matter in the absence of nematodes, and therefore organic matter rich soil boost their existence [75].

#### 7.1.1 Arthrobotrys oligospora

*A. oligospora* is an important and most common nematophagous fungus. This fungus traps nematode juveniles facultatively for its nitrogen requirement. It also obtains carbon and energy-rich foods from dead and decomposed organic matter [77]. *In vitro* experiment shows the high efficiency of *A. oligospora* in the killing and capturing of *M. incognita* J2s. The microscopic study also revealed that the J2s of RKNs were trapped by adhesive loops of *A. oligospora* [78]. Moreover, *A. oligospora* treatment significantly reduces the disease development caused by RKNs on tomato plants and shows 74 % predation against *M. incognita* in comparison to 36 % of the control (having only sterilized distilled water without *A. oligospora*) [79].

#### 7.1.2 Drechmeria coniospora

Fungus *D. coniospora* infects nematodes with their spores (zoospores or conidia) via the attachment of conidia to the nematode cuticle and inserting the hyphae in nematode epidermis and killing them [80]. However, conidia attach to various PPNs such as *Cephalenchus* sp., *Pratylenchus penetrans*, *Heterodera schachtii,* and *Ditylenchus* spp. but the attachment of spores to any nematode species does not favour its killing and infection [76,80].

#### 7.1.3 *Pochonia* chlamydosporia, *Paecilomyces lilacinus* and *Lecanicillium psalliotae*

*P. chlamydosporia*, *P. lilacinus*, and *L. psalliotae* are considered as potent BCAs among all female and egg parasitic fungi of RKNs and cyst nematode. These fungi mainly attack nematode females and their eggs because these are the main targets. However, J2s of RKNs inside egg masses can also be attacked by *P. chlamydosporia* [81,82]. A few strains of *P. chlamydosporia* also stimulate the defence in tomato plants by activation of the salicylic acid (SA) pathway against *M. incognita*. It has been observed that co-inoculation of both fungus and nematode in tomato plants revealed the expression of defence-related protein 1 (PR1), lipoxygenase (LoxD) genes, pathways *viz*., jasmonic acid (JA) and SA pathways which provide resistance against *M. incognita*. In this co-inoculation experiment, fungal chlamydospores were inoculated in the soil just one week before the inoculation of *M. incognita* J2s [83]. *P. lilacinus* is another nematode egg parasitic fungus, used as BCAs against RKNs [28]. According to Kiewnick and Sikora [84], inoculation of *P. lilacinus* strain 251 (PL251) in tomato field infested with *M. incognita* reduces the egg masses by 74 %, root galling by 66 % and the population of nematodes by 71 % compared to untreated control. However, *P. lilacinus* directly infect eggs and sedentary stages of nematodes by producing few nematicidal proteins *viz*., chitinases, leucinotoxins, acetic acid, and proteases [85].

#### 7.1.4 *Pleurotus ostreatus*

**Fig. 3:**
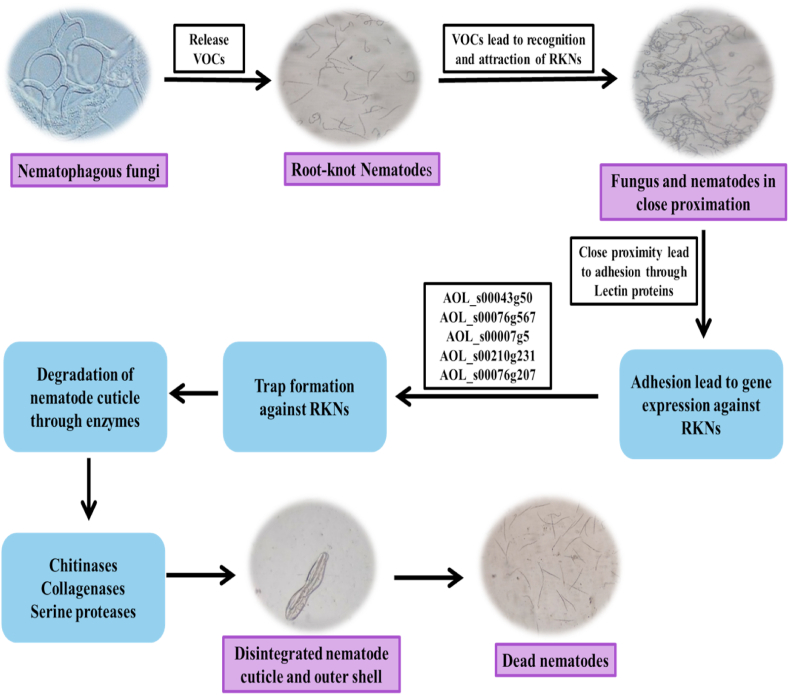
Modes of action of nematophagous fungi as bio-control agents against root-knot nematodes on agricultural crops.

*P. ostreatus* (oyster mushroom) as a toxin-producing fungus having the ability to kill nematodes by paralyzing them. This fungus feeds on nematodes for their N_2_ requirement under low nutrient conditions. However, current studies show that *P. ostreatus* kill nematodes by increasing calcium (Ca) influx and necrosis in the neuromuscular system of *Caenorhabditis elegans* through nematode sensory cilia [86,87]. A few studies also revealed the bio-control efficiency of *P. ostreatus* against PPNs including root-knot nematode, *M. incognita* and sugar beet nematode, *Heterodera schachtii* in cowpea [88,89] (Fig. 3).

### 7.2 *Trichoderma* spp.

*Trichoderma* spp. classified as cosmopolitan, saprotrophic, and widely applicable fungus. Consider as dominant among all known bio-control fungi against RKNs, and colonize the plant-soil ecosystems. They have ability to protect plants from RKNs and other soil-borne pathogens by colonizing the rhizosphere [90]. They grow easily and are propagated on synthetic media under lab conditions, improving plant growth and inducing resistance against RKNs [91]. They enhance the nutrient uptake from the soil in the inoculated plant [92]. *Trichoderma* spp. also prevents nematode penetration by parasitizing the nematode egg shell and cuticle through its conidia and improves plant growth as well [93]. The attachment affinities to *Meloidogyne* spp. eggs, cuticle, or gelatinous matrix of egg masses are species-specific [94]. The most effective bio-control properties are mainly attributed to the different species *viz*., *T. viride*, *T. citrinoviride* (Snef1910 strain), *T. virens*, *T. harzianum* (strain MZ025966), *T. koningii*, *T. longibrachiatum*, *T. asperellum*, and *T. polysporum* which have a significant impact on disease development caused by RKNs.

#### 7.2.1 Modes of action of *Trichoderma* spp.

In addition to competition for food and shelter with RKNs, *Trichoderma* spp. also shows antagonistic activity against harmful fungi [95] possessing a high ability to adapt to different environmental conditions and reducing the effect of harmful pathogens by their fast-growing rate [96,97]. However, the current knowledge about the *Trichoderma* induced resistance in plants against other biotic pathogens *viz*., bacteria, fungi, and viruses are unknown [91]. Although, previous researches find out that *Trichoderma* mediated resistance in plants may play an impressive role in reducing the infection caused by RKNs and other harmful pathogens [98,99]. Root colonization by *Trichoderma* spp. changes the metabolism and physiology of the host plant, resulting in the production of several secondary metabolites (SMs) that function as defense responses against RKNs [100,101].

#### 7.2.2 *Trichoderma* spp. alleviates root-knot nematodes-induced oxidative stress

Reactive oxygen species (ROS) are continuously formed in plants but RKN infection increases ROS production [101,102]. Excessive production of ROS at infection sites harms the cell membranes by membrane lipid peroxidation [101,103]. However, low levels of Malondialdehyde (MDA) and electrolyte leakage by 30.85 % and 38.89 % were found in tomato roots treated with *T. herzianum* and RKNs simultaneously, compared with RKNs infested roots [104]. To normalize cellular functions, it is necessary to keep the ROS below the threshold level by promoting antioxidant production, and acting as a ROS scavenger [102,105].

#### 7.2.3 *Trichoderma* spp. increases the concentrations of secondary metabolites

Secondary metabolism plays an important role in the production of antioxidants and SMs required for defence and to lower the high level of ROS under unfavourable conditions. Importantly, several SMs *viz*., phenols, flavonoids, lignin, and cellulose act as antioxidants to increase resistance and protect host plants from RKNs infection [100,106,107]. Yan et al. [104], found an increased level of flavonoids, cellulose and lignin in *T. herzianum* treated tomato plants infested with RKNs compared to the untreated plants. Ahmad et al. [71] also finds that the combined application of *T. harzianum* with fly ash improved the SMs profile and defence-related genes in chili (*Capsicum annum*) against RKNs.

#### 7.2.4 Trichoderma enhances the enzymatic activity and transcript levels of genes involved in secondary metabolism

*Trichoderma harzianum* treated plants showed an increased level of enzymes that participated in secondary metabolisms to provide resistance against PPNs. Key enzymes such as cinnamyl alcohol dehydrogenase (CAD), glucose-6-phosphate dehydrogenase (G6PDH), phenylalanine ammonia-lyase (PAL), polyphenol oxidase (PPO), shikimate dehydrogenase (SKDH), guaiacol peroxidase (G-POD), 4-coumarate CoA ligase (4CL), and caffeic acid peroxidase (CA-POD) involved in secondary metabolisms in treated tomato plants compared to untreated control [104]. Many studies also suggest the importance of a few genes *viz*., CAD, cinnamate-4-hydroxylase (C4H), and PAL in the regulation of the plant defence and phenylpropanoid pathway against RKNs [108,109]. Pathogenesis-related proteins (PRPs), such as peroxidases, chitinases, and β-1,3–glucanase shows nematicidal and antimicrobial activity [99,110,111]. *In vitro* observations suggest these enzymes damage the major parts of the nematode cuticle, and eggshell and ultimately lead to death [112].

#### 7.2.5 *Trichoderma* spp. promotes the content of jasmonic acid (JA) and salicylic acid (SA)

To obtain good results and insight into the mechanisms of *Trichoderma*-induced resistance against RKNs, it is necessary to modify the SA and JA contents in the tomato roots by inoculating *Trichoderma* in the roots. However, a high level of both SA and JA was found in RKN-infected plant roots compared to non-infected plants (Table 3).

**Table 3:** Modes of action of fungal species and their strains against root-knot nematodes on agricultural crops.

Fungal species and their strains	Modes of action	Target root-knot nematode species	Test crop	Type of study	Authors
*Pochonia chlamydosporia*	Activation of the salicylic acid pathway in plant	*Meloidogyne incognita*	Tomato	*In vivo*	[29]
*Trichoderma longibrachiatum*	Activation of protease enzyme in plant	*M. incognita*	Cucumber	*In vitro, in vivo*	[113]
*Arthrobotrys oligospora*	Formation of traping structure *viz*., adhesive loops of hyphae	*M. incognita*	Tomato	*In vitro, in vivo*	[79]
*T. viridae* and *T. herzianum*	Direct inhibition of nematode	*M. incognita*	Tomato	*In vivo*	[18]
*Penicillium chrysogenum* strain Snef1216	Direct inhibition of the reproduction rate of nematode	*M. incognita*	Cucumber	*In vivo*	[114]
*P. chlamydosporia*	Direct inhibition of nematode	*M. incognita*	Carrot	*In vivo*	[150]
Combined application of *T. hammatum* with *Paecilomyces lilacinus* and extract of *Tegetus erecta*	Direct inhibition of nematode and their life cycle	*M. javanica*	Tomato	*In vivo*	[115]
*Purpureocillium lilacinum*	Direct inhibition of nematode and their life cycle	*M. incognita*	Tomato	*In vivo*	[116]
*Mortierella globalpina*	Trapping, adhesion, and penetration to nematode cuticle through hyphae	*M. chitwoodi*	Tomato	*In vitro, in vivo*	[117]
*Scutellospora heterogama*	Root colonization and competition for space and nutrients with nematode	*M. incognita*	Passion fruit (*Passiflora alata*)	*In vivo*	[118]
*Myrothecium verrucaria* strain X-16	Parasitizes eggs, second-stage juveniles of nematodes	*M. hapla*	Cucumber	*In vivo*	[119]
*Lecanicillium muscarium*	Parasitization of eggs, second-stage juveniles, and females of nematodes	*M. incognita*	Tomato	*In vitro, in vivo*	[120]
Combined application of *P. chrysogenum* and *P. chlamydosporia*	Reduction in egg hatching, gall indices, and egg masses, increase juveniles' mortality	*M. incognita*	Cucumber	*In vitro, in vivo*	[121]
*A. thaumasia* and *Tolypocladium cylindrosporum*	Increase parasitism of nematodes and reduction in galls, egg masses	*M. incognita* and *C. elegans*	Tomato	*In vitro, in vivo*	[122]
*P. lilacinum*	Inhibition of disease and life cycle of the nematode	*M. incognita*	Mung bean	*In vivo*	[123]

### 7.3 Advantages of *Trichoderma* spp. other than bio-control mechanisms

**Fig. 4:**
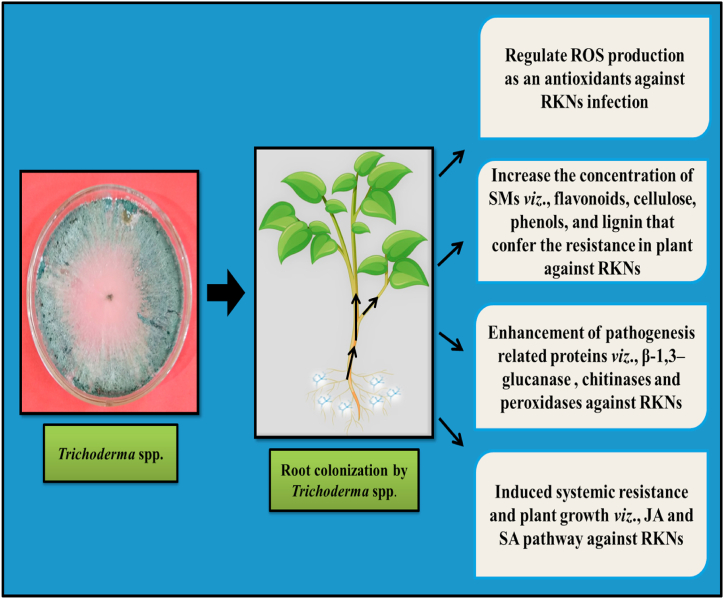
Modes of action of *Trichoderma* spp. as bio-control agent against root-knot nematodes on agricultural crops.

Besides BCAs, *Trichoderma* spp. as a part of soil microbes, play a key role in the decomposition, and decontamination/removal of harmful substances, i.e., xenobiotic from the soil ecosystem. Decontamination/removal of harmful substances is considered as ‘bio-remediation’ involving the use of microbes to change the toxic compounds into non-toxic while decomposition leads to the release of beneficial nutrients available for plants growth [124,125]. According to Weber et al. [126], *T. viride* uses nitrogenous compounds, *viz*., (trinitrotoluene, TNT explosive) at 100 and 50 ppm doses to fulfil their nitrogen requirement for normal growth and development of plants. Besides, the utilization of nitrogenous explosives as a nitrogen source, *Trichoderma* spp. also has the ability to degrade unwanted hydrocarbons from the aquatic ecosystem and bio-remediate them [127]. Few other researches also reported the use of *T. harzianum* strain T22, for the biodegradation of diesel fuel, allowing it to be used as a source of carbon [128]. It has been found that the combined application of chemical fertilizers and *Trichoderma* into the soil increases plant productivity, nutritional quality, and reproductive and vegetative development. The overall role and application of different species of *Trichoderma* make it as potent BCAs, and soil nutrient mobilizer, improving the yield and quality of the crops (Fig. 4).

## 8 Bacteria-mediated bio-control against root-knot nematodes

Bacteria are microscopic organisms that grow in diverse environmental conditions, ranging from radioactive waste, hot water springs, soil, and the deep biosphere of the earth's crust [[129], [130], [131]]. They also exist as symbionts and parasites on several plants and animals. Some bacterial genera *viz*., *Bacillus*, *Pseudomonas*, *Serratia*, *Pasteuria*, *Burkholderia*, *Achromobacter*, *Arthrobacter*, and *Rhizobium* possess nematicidal action and are considered as potent BCAs of RKNs. Few commercial products have also been obtained from bacteria that possess nematicidal potential [74]. Besides BCAs of nematodes, bacteria also enhance plant growth by increasing nutrient uptake from the soil.

The whole bio-control mechanisms depend on the competition for food and space with nematodes, the production of antibiotic compounds and hydrolytic enzymes against nematodes, and the induction of systemic resistance (ISR) in host plants. Among these, antibiosis is the most widely used mechanism involving the production of volatile organic compounds (VOCs), toxins, and some antibiotics against nematodes.

### 8.1 Modes of action

#### 8.1.1 Antibiosis

It involves the production of toxic compounds by microorganisms harmful to other microbes. During the trophophase of the cell cycle, the development of bacterial cells is ideally higher however the metabolites and antibiotic compounds are formed during the idiophase when the nutrients become drained and the cell has expanded extensively [132]. Such compounds are beneficial for plant growth and development.

#### 8.1.2 Rhizospheric competition

Competition for food and space between pathogenic and non-pathogenic microorganisms in the rhizosphere is known as rhizospheric competition. Rhizospheric competence also promotes the population of beneficial microbes in the vicinity of plant roots. However, these beneficial soil microbes protect plants by root colonization and deplete all the resources from the rhizosphere that are available and accessible to RKNs' growth and development. Such an important relationship between microbes and plants found in the rhizosphere enhances plant health or helps the plant overcome abiotic or biotic stress [133].

#### 8.1.3 Production of hydrolytic enzymes

Production of hydrolytic enzymes by rhizobacteria is also an important approach to controlling RKNs and other harmful pathogens effectively. Few *Bacillus* spp. produces various types of enzymes and enzyme complexes *viz*., chitinases, proteases, lipases, and collagenases. These enzymes affect various stages of PPNs and kill them. Chitinases enzyme produced by the bacterial species, *B. pumilus*, *Serratia marcescens*, and *B. subtilis* degrade cuticle and the outer surface of eggs and J2s of RKNs [134,135]. Collagenases are another potent lytic enzyme produced by the *B. cereus* against the J2s of *M. javanica* [134]. Some other groups of lytic enzymes like Glucanases, cellulases, and pectinases produced by *Pseudomonas* against *M. incognita* [134].

#### 8.1.4 Induced systemic resistance (ISR)

ISR is one of the possible modes of action that induces resistance in host plants by activating the nematicidal compounds and preventing infection by nematodes. Several bacterial genera like *B. pasteurii*, *B. subtilis*, *B. cereus*, *B. pumilus*, *B. amyloliquefaciens*, *P. putida*, *S. marcescens*, *P. fluorescence*, and *Rhizobium leguminosarum* can provoke ISR [136]. ISR against *M. javanica* and *M. graminicola* by fluorescent pseudomonads has been well documented [137,138]. The antibiotic compound 2,4-diacetylphloroglucinol (DAPG) found as a systemic resistance-inducing agent in plants [139,140]. However, VOCs and cyclic lipopeptides are also the key activators of ISR by endospore-forming gram-positive bacteria.

### 8.2 Bacterial species and their modes of action

#### 8.2.1 *Bacillus* spp.

Several studies suggested that *Bacillus* spp. acts as an important bio-pesticide to control RKNs and improve plant growth. Antinemic action of *Bacillus* spp. based on their enzymes, toxic proteins, and antibiotic compounds they possess for example, antibiotics production by *B. subtilis*, and *B. cereus*, toxic proteins production namely Cry proteins by *B. thuringiensis* and enzymatic action by *B. firmus* [141]. According to Li et al. [142], the application of *B. cereus* (strain BCM2) in tomato plants reduces the damage and population of *M. incognita* by 67.1 % compared to untreated plants. Few other strains *viz*., *B. cereus* (strain Jdm1) enhance the growth of tomato plants by inhibiting egg hatching, root galling (43 %), and the number of *M. incognita* J2s *in vivo* [143]. *B. pumilus* (strain L1) is another example with anti-RKNs potential, produces chitinases and protease enzymes that degrade and digest the cuticle of *M. arenaria* [144]. Culture supernatant and crude extract of *B. amyloliquefaciens* (strain Y1) were evaluated to control *M. incognita* by inhibiting egg hatching and increasing J2s mortality both *in vivo* and *in vitro* on tomato plants [145].

Bio-control of RKNs and other PPNs attributed to *B. thuringiensis* (Bt) that includes the formation of Cry protein or crystal protein (proteinaceous protoxin crystals). It has been found that several crystal proteins obtain from transgenic plants and their application to protect plants from RKNs [78]. Presently 3 families of crystal proteins like Cry5, Cry6, and Cry55 cause the killing and growth retardation of nematode J2s [146]. Cry5 is the most effective and potent crystal protein among all Cry proteins that causes the lysis of intestinal tissues of nematodes.

#### 8.2.2 *Pasteuria penetrans*

**Fig. 5:**
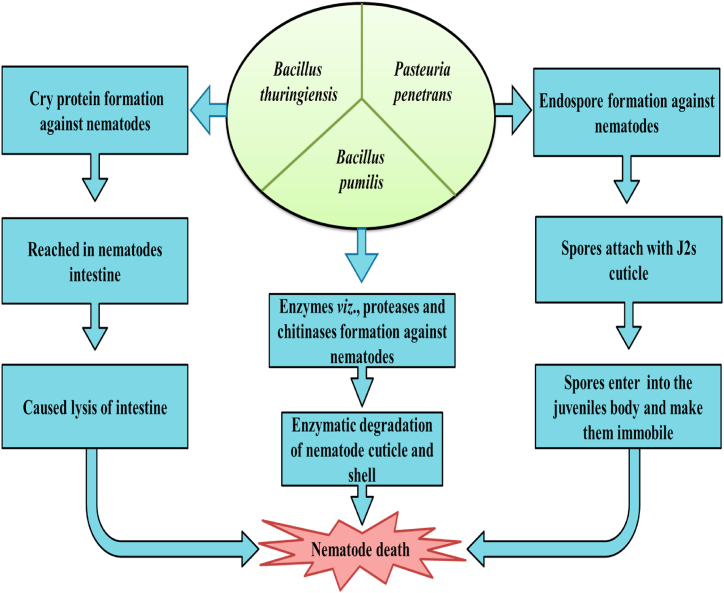
Modes of action of bacteria as bio-control agents against root-knot nematodes on agricultural crops.

*Pasteuria penetrans* is an endospore-forming bacterium, that parasitizes the J2s of RKNs [147]. J2s with bacterial spores become immobile and unable to penetrate the roots [147]. Bacterial spores attached to the nematode's cuticle, enter the body by germination tube, form endospores inside, and finally lead to nematode death (Fig. 5; Table 4).

**Table 4:** Modes of action of bacterial species and their strains against root-knot nematodes on agricultural crops.

Bacterial species and their strains	Modes of action	Target root-knot nematode species	Test crop	Type of study	Authors
*Bacillus amyloliquefaciens* (strain Y1)	Production of antinemic compounds dipeptide cyclo (D-Pro-L-Leu)	*M. incognita*	Tomato	*In vitro*, *in vivo*	[145]
*B. cereus* (strain Jdm1)	Inhibited the J2s activity, egg hatching, and number of galls	*M. incognita*	Tomato	*In vitro*, *in vivo*	[143]
*B. velezensis* (strain Bv-25)	Inhibition of egg hatching and suppression of genes ord-1, mpk-1, and flp-18 of *M. incognita*	*M. incognita*	Cucumber	*In vitro*, *in vivo*	[148]
*B. megaterium*	Reduction in nematode population, number of galls, and egg masses	*Meloidogyne* spp.	Sugar beet	*In vivo*	[149]
Combined application of *Pseudomonas fluorescens* (strain CHA0) with Ammonium molybdate	Kill nematodes and inhibit their penetration in the mung bean	*M. javanica*	Mung bean	*In vitro*	[150]
*B. pumilus* (strain L1)	Killing and degradation of nematodes by protease and chitinase enzymes	*M. arenaria*	Tomato	*In vitro, in vivo*	[144]
*B. cereus* (strain BCM2)	The killing of nematodes by repelling juveniles from plants	*M. incognita*	Tomato	*In vivo*	[142]
Application of *B. cereus*, *B. licheniformis*, *Lysinibacillus sphaericu*, *P. fluorescens* and *P. brassicacearum*	Inhibition of the reproductive cycle of the nematode	*M. incognita*	Tomato	*In vivo*	[151]
*P. fluorescens* (strain Pf1)	Activation of defense enzymes *viz*., phenol, peroxidase (PO), polyphenol oxidase (PPO), phenyl ammonia lyase (PAL), and chitinase	*M. graminicola*	Rice	*In vitro*	[152]
*B. megaterium*	Reduction in nematode penetration, gall formation, and egg hatching	*M. graminicola*	Rice	*In vitro*, *in vivo*	[153]
Application of *B. firmus* T11, *B. aryabhattai* A08, *Paenibacillus barcinonensis* A10, *Paenibacillus alvei* T30, and *B. cereus*	Paralysis of second-stage juveniles	*M. incognita*	Tomato and Carrot	*In vitro*	[154]
Five isolates of *Bacillus*	Causes juveniles mortality and reduction in galls	*M.javanica* (Chitwood)	Soybean	*In vitro*, *in vivo*	[155]

### 8.3 Role of bacteria in plant growth promotion other than bio-control mechanisms

Plant growth-promoting bacteria (PGPB) includes a diverse group of microorganisms that represent a broad range of genera. Few genera *viz*., *Pseudomonas*, *Bacillus*, *Lactobacillus*, and actinobacteria are involved in plant growth promotion [[156], [157], [158], [159]]. They perform growth promotion, advancement, and development of plants by increasing beneficial microbiota in the vicinity of the rhizosphere, root colonization, competition with native harmful microbes, and build-up resistance in host plants against RKNs [160]. Furthermore, *Herbaspirillum*, *Azospirillum*, *Acetobacter*, *Serratia*, *Paenibacillus*, *Burkholderia*, and *Rhodococcus* have also been found to increase crop growth and production [161]. According to Chakraborty et al. [162], rhizobacterial strains *viz*., *B. pumilis* and *B. amyloliquefaciens* demonstrated *in vitro* plant growth-promoting attributes, like siderophores production, phosphate solubilization, Indole-3-acetic acid (IAA) production and antagonisms against RKNs. Such strains also favour an increment in the growth and yield of tea crops in terms of root and shoot biomass and the number of leaves in the field conditions [162]. Siderophores production by plants and bacterial strains under low iron (Fe) conditions is also an important plant growth-promoting factor [163,164]. Besides siderophores, IAA is also the most studied auxin formed by PGPR plays a key role in plant growth by involving in plant–microbe interactions, changes in the transcriptional hormones, cell wall-related and defence-related genes [165,166]. It also induces root biomass, reduction in stomatal density and size to prevent water loss, and activates auxin response genes to improve plant growth [167,168].

## 9 Conclusions and future outlooks

Agriculture is an important part of the economy in many countries. Most of the human population depends on crops for their food. However, several crops (vegetables and fruits) are severely attacked by RKNs. When evaluating the significant damage caused by RKNs and the limitations made by the government for the use of pesticides, it is necessary to scientists and researchers to invent novel eco-friendly approaches to control RKNs. According to previous studies on nematode management, it has been found that the excessive use of chemicals polluted the environment, soil, and water, and also influences human health. Therefore, by considering these all problems, microbial bio-control emerged as a potent substitute for chemicals.

Several microbes such as fungi, and bacteria with their different strains have great nematicidal action. Fungi possess various modes of action against nematodes such as digestion and absorption of nematode cuticle and their penetration, rhizospheric colonization, and induction of resistance in plants. Whereas, bacteria counter-attack the RKNs parasitism through antibiosis (production of toxic compounds), rhizospheric competition with nematodes, production of several lytic enzymes (*viz*., collagenases, proteases, lipases, and chitinases), and induction of systemic resistance. Besides bio-control mechanisms, fungi, and bacteria also enhances plant growth and yield by increasing nutrient (N and P) uptake from the soil and nitrogen fixation as well.

Bio-control of RKNs has been around for the last two decades, but it is unable to achieve better results and more attention as new species are identified and examined as bio-control agents against RKNs. Currently, scientists are engaged in RNA sequencings involving 16S and 18S rDNA sections, having great potential for the detection of new BCAs against RKNs. This approach will make bio-control studies faster, cheaper, more effective, and more practical in the future. Furthermore, it would be also helpful to focus on environmental health, soil fertility, and non-target microbial diversity during nematode management.

## Ethical approval

Not applicable.

## Consent to participate

Not applicable.

## Consent to publish

Not applicable.

## Funding

This study was finnacillay supported by Aligarh Muslim University (India), National Research Institute for Agronomic Research (INRA, Morocco), and the Phytopathology Unit of the Ecole Nationale d'Agrioculture de Meknès under SIRAM project (ENA-Meknes, Morocco).

## Availability of data and materials

Not applicable.

## CRediT authorship contribution statement

**Amir Khan:** Conceptualization, Data curation, Formal analysis, Methodology, Writing – original draft. **Mohammad Haris:** Conceptualization, Data curation, Formal analysis, Methodology, Writing – original draft. **Touseef Hussain:** Conceptualization, Data curation, Formal analysis, Funding acquisition, Methodology, Project administration, Supervision, Writing – original draft, Writing – review & editing. **Abrar Ahmad Khan:** Conceptualization, Data curation, Formal analysis, Methodology, Writing – original draft, Writing – review & editing. **Salah-Eddine Laasli:** Conceptualization, Data curation, Formal analysis, Investigation, Writing – review & editing. **Fouad Mokrini:** Conceptualization, Formal analysis, Writing – review & editing. **Rachid Lahlali:** Conceptualization, Data curation, Formal analysis, Funding acquisition, Methodology, Supervision, Writing – review & editing.

## Declaration of competing interest

The authors declare that they have no known competing financial interests or personal relationships that could have appeared to influence the work reported in this paper.

## Abbreviations

PPNs: Plant-parasitic nematodes
RKNs: Root-knot nematodes
J1s: First stage juveniles
J2s: Second stage juveniles
GCs: Giant cells
CWDEs: Cell wall digestive enzymes
SA: Salicylic acid
JA: Jasmonic acid
ETI: Effector triggered immunity
PTI: Pathogen triggered immunity
INM: Integrated nematode management
BCAs: Bio-control agents
PRP: Pathogenesis related proteins
ROS: Reactive oxygen species
SMs: Secondary metabolites
ISR: Induced systemic resistance
VOCs: Volatile organic compounds
